# Epidemiology and burden of influenza in healthy children aged 6 to 35 months: analysis of data from the placebo arm of a phase III efficacy trial

**DOI:** 10.1186/s12879-019-3920-8

**Published:** 2019-04-04

**Authors:** Clotilde El Guerche-Séblain, Annick Moureau, Camille Schiffler, Martin Dupuy, Stephanie Pepin, Sandrine I. Samson, Philippe Vanhems, François Schellevis

**Affiliations:** 1 Global Vaccine and Epidemiology Department, Sanofi Pasteur, Lyon, France; 2 Global Clinical Biostatistics Department, Sanofi Pasteur, Marcy l’Étoile, France; 3 Global HEOR Department, Sanofi Pasteur, Lyon, France; 4 Global Biostatistics Department, Sanofi Pasteur, Marcy l’Étoile, France; 5 Global Clinical Sciences, Sanofi Pasteur, Marcy l’Étoile, France; 6 Global Medical Affairs, Sanofi Pasteur, Lyon, France; 7 Epidemiology and International Health Team, Emergent Pathogens Laboratory, Fondation Mérieux, International Center for Research in Infectiology, National Institute of Health and Medical Research, U1111, National Center of Scientific Research, Mixed Scientific Unit 5308, École Nationale Supérieure de Lyon, Université Claude Bernard Lyon 1, Lyon, France; 8 Netherlands Institute for Health Services Research, Utrecht, The Netherlands; 9 Department of General Practice & Elderly Care Medicine, Amsterdam Public Health Research Institute, Amsterdam University Medical Centres, Amsterdam, The Netherlands

**Keywords:** Influenza, Children, Fever, Hospitalisation, Epidemiology, Clinical trial, Acute otitis media, Acute lower respiratory infection, Pneumonia, Antibiotic use

## Abstract

**Background:**

Despite World Health Organization recommendations, in many countries young children are not targeted for influenza vaccination. To help inform influenza vaccination policy, we examined the occurrence and burden of influenza in healthy children aged 6 to 35 months using data from a recent phase III placebo-controlled influenza vaccine trial conducted in countries in the Northern and Southern Hemispheres.

**Methods:**

This was an analysis of data from participants included in the placebo arm of a phase III clinical trial in healthy children aged 6 to 35 months (EudraCT no. 2013–001231-51). Included children had never been vaccinated for influenza and were observed for one influenza season. Outcome measures included the occurrence of influenza-like illness (ILI), laboratory-confirmed influenza, virus types/subtypes, severe symptoms and complications of confirmed influenza, and healthcare use associated with confirmed influenza.

**Results:**

Data from 2210 participants were analysed. ILI was reported for 811 participants (36.7%). Of these, 255 participants (31.4%) had 263 virologically confirmed episodes of influenza. The overall influenza attack rate was 11.5%. The most common influenza virus detected was A(H3N2) (40.7%), followed by B/Yamagata (23.6%), A(H1N1) (18.6%), and B/Victoria (8.0%). Grade 3 fever was reported in 24.3% of confirmed episodes, acute lower respiratory infection in 8.7%, acute otitis media in 6.1%, and pneumonia in 1.9%. In most influenza episodes (93.2%), antipyretics, analgesics, or non-steroidal anti-inflammatory drugs were taken. Antibiotics were prescribed for 41.4% of influenza episodes. More than half of the influenza episodes (57.0%) resulted in outpatient visits. Influenza resulted in overnight hospitalisation in 1.1% of episodes.

**Conclusions:**

Influenza is associated with a significant burden of disease in healthy children. This analysis also revealed that antibiotics continue to be frequently used for young children with influenza.

**Trial registration:**

EudraCT no. 2013–001231-51.

**Electronic supplementary material:**

The online version of this article (10.1186/s12879-019-3920-8) contains supplementary material, which is available to authorized users.

## Background

Influenza causes significant morbidity in young children and, each year, leads to hospitalisation of approximately 870,000 children under 5 years of age worldwide [1]. Whereas annual influenza attack rates range from 5 to 10% in adults, they range from 20 to 30% in children [2]. Young children are also important vectors for the spread of influenza in the community and within families because they are in close contact with each other in schools and day care, have close contact with adults and the elderly, have poor hygiene habits, have limited pre-existing immunity, and shed high virus titres [3].

The World Health Organization (WHO) recommends that children 6 to 59 months of age be considered a priority for influenza vaccination, along with pregnant women, elderly adults, individuals with specific chronic medical conditions, and healthcare workers [4]. Despite these recommendations, many countries do not have vaccination policies, and those that do often do not include young children as a target group [5]. This may be because severe influenza has been thought to be a problem limited to children with underlying high-risk conditions [6]. More information about the burden of influenza in young children is therefore needed to inform and support influenza vaccination policies.

A recent phase III placebo-controlled clinical trial conducted in several countries in the Northern and Southern Hemispheres examined the efficacy, immunogenicity, and safety of an inactivated quadrivalent influenza vaccine in healthy children aged 6 to 35 months [7]. To provide additional understanding of the occurrence and burden of influenza in healthy young children, we examined data collected from participants randomised to the placebo arm of the trial. This analysis focused on the occurrence of laboratory-confirmed influenza, the virus types/subtypes, severe symptoms and complications of influenza, and influenza-related healthcare use.

## Methods

### Study design

This was an analysis of data from participants included in the placebo arm of a phase III randomised controlled clinical trial (EudraCT no. 2013–001231-51; registered March 13, 2014) [7]. The participant flow diagram for the original study is provided in Additional file 1: Figure S1. Participants were included during the 2014 influenza season in South Africa, the 2014 and 2015 influenza seasons in the Philippines, the 2014/2015 influenza season in Honduras and the Dominican Republic, and the 2014/2015 and 2015/2016 influenza seasons in France, Greece, Italy, Spain, and Romania. Healthy children aged 6 to 35 months with no history of influenza vaccination were randomised to receive two placebo injections or two full doses 28 days apart of quadrivalent split-virion inactivated influenza vaccine, the licensed trivalent split-virion inactivated vaccine, or an investigational trivalent influenza vaccine that had the recommended B strain replaced with one from the other of the two influenza B lineages.

The primary endpoints for the current analysis were the occurrence of an influenza-like illness (ILI) and the occurrence of laboratory-confirmed influenza by virus type/subtype. Other endpoints for this analysis included the occurrence of acute otitis media (AOM), acute lower respiratory infection, outpatient visits, inpatient hospitalisation, grade 3 fever, and grade 3 ILI symptoms. ILI, laboratory-confirmed influenza, and influenza virus subtypes were assessed as described previously [7]. Participants were considered to have ILI if they had a fever ≥38 °C lasting ≥24 h concurrently with cough, nasal congestion, rhinorrhoea, pharyngitis, otitis, vomiting, or diarrhoea. For subjects aged < 24 months, grade 1 fever was defined as a temperature ≥ 38.0 °C to ≤38.5 °C, grade 2 fever as > 38.5 °C to ≤39.5 °C, and grade 3 fever as > 39.5 °C [7]. For subjects aged ≥24 months, grade 1 fever was defined as a temperature ≥ 38.0 °C to ≤38.4 °C, grade 2 as ≥38.5 °C to ≤38.9 °C, and grade 3 as > 39.0 °C. ILI symptoms were considered grade 1 if they did not interfere with daily activity, grade 2 if they caused some interference with daily activity, and grade 3 if they were significant and prevented daily activity.

Temperature and ILI symptoms were recorded by parents or legal representatives on diary cards. From 14 days after the second placebo injection until the end of the influenza season, parents or legal representatives of participants were instructed to contact their study site within 24 h if their child experienced ILI symptoms. In addition, during the peak of the influenza season, investigators contacted the parents or legal representatives of participants twice each week by phone to ask about possible ILI symptoms. If ILI was suspected during a phone call, the investigator systematically collected information on symptoms and healthcare utilisation and arranged for the participant to visit the study site within 10 days of ILI onset. For all suspected ILIs, information on ILI symptoms and healthcare utilisation were updated through follow-up phone calls scheduled 15 days after onset and subsequently every 30 days until full recovery from ILI. In the arranged visits for suspected ILI, a nasopharyngeal swab was taken for laboratory confirmation of influenza by both viral culture and reverse transcription polymerase chain reaction. Sanger sequencing of the hemagglutinin and neuraminidase full-gene segments was performed to identify the specific type or subtype of influenza. Healthcare utilisation and the presence of AOM and acute lower respiratory infection were also recorded during the visit. For healthcare utilisation, inpatient hospitalisation was defined as a hospital admission resulting in an overnight stay, and an outpatient visit was defined as an unscheduled ambulatory visit with a physician or other healthcare professional without overnight stay. AOM was defined as a visually abnormal tympanic membrane suggesting an effusion in the middle ear cavity concomitant with one or more of the following symptoms: fever (≥ 38 °C), earache, irritability, diarrhoea, vomiting, acute otorrhoea not caused by external otitis, or other symptoms of a respiratory infection. Acute lower respiratory infection was defined as radiologically confirmed pneumonia, bronchiolitis, bronchitis, or laryngotracheobronchitis [7, 8].

### Statistical analysis

Statistical analyses were descriptive only and were performed using SAS version 9.2 (SAS Institute, Cary, NC, USA). Attack rates were calculated using the number of influenza positive cases, with the denominator as the number of included patients in the placebo arm. This corresponds to the incidence per influenza season. The 95% confidence intervals of the attack rates were calculated using a normal approximation. Occurrence of grade 3 symptoms, complications, and healthcare use were calculated using confirmed influenza episodes as the denominator.

## Results

### Participants

This analysis included 2210 of the 2591 participants randomised in the placebo arm of the trial who completed the two-dose vaccination schedule and had at least one contact during follow-up (1469 from the Philippines, 502 from the included European countries, and 239 from South Africa) [7]. Participants from Honduras and the Dominican Republic (*n* = 379) were excluded from this analysis because they had been included outside of the influenza season, resulting in no influenza cases detected [7]. Two participants randomised to the placebo arm were excluded from this analysis because they had accidentally received one dose of influenza vaccine instead of placebo. The overall mean age was 19.9 months, and mean ages were similar in each region (Table 1). Overall, the male/female ratio was 1.1, and more boys than girls were included in all regions.

**Table 1:** Characteristics of participants included in the analysis

	South Africa	Philippines	European countries ^a^	Overall
Characteristic	*N* = 239	*N* = 1469	*N* = 502	*N* = 2210
Male/female ratio	1.37	1.05	1.03	1.10
Age (months), mean ± standard deviation	19.7 ± 7.9	19.9 ± 8.6	20.0 ± 8.2	19.9 ± 8.4

^a^France, Greece, Italy, Spain, and Romania

### ILI and laboratory-confirmed influenza

ILI was reported by 811 (36.7%) of the 2210 participants (Table 2). Of these, 255 participants had 263 virologically confirmed episodes of influenza. The influenza attack rate was more than two times higher in the European countries (20.5%) than in South Africa (6.7%) and the Philippines (9.3%). The overall influenza attack rate was 11.5%.

**Table 2:** Proportion of participants with influenza-like illness and laboratory-confirmed influenza, and influenza attack rates

	South Africa	Philippines	European countries ^a^	Overall
Measure	*N* = 239	*N* = 1469	*N* = 502	*N* = 2210
Influenza-like illness, n (%) ^b^	45 (18.8)	510 (34.7)	256 (51.0)	811 (36.7)
Laboratory-confirmed influenza, n (%) ^c^	16 (35.6)	136 (26.7)	103 (40.2)	255 (31.4)
Influenza attack rate, % (95% CI) ^b^	6.7 (3.5; 9.9)	9.3 (7.8; 10.7)	20.5 (17.0; 24.1)	11.5 (10.2; 12.9)

Abbreviations: *CI*, confidence interval

^a^France, Greece, Italy, Spain, and Romania

^b^Percentage of all participants

^c^Percentage of participants with influenza-like illness

The most common serotype was A(H3N2) (40.7% of confirmed episodes), followed by B/Yamagata (23.6%) and A(H1N1) (18.6%). Relatively few episodes of B/Victoria infection were reported (8.0%) (Table 3). Attack rates were 2.2% for A(H1N1), 4.6% for A(H3N2), 2.8% for B/Yamagata and 1.0% for B/Victoria. By region and season, A(H3N2) was the most frequently detected influenza subtype during the 2014 season in South Africa, the 2015 season in the Philippines, and the 2014/2015 season in the included European countries, whereas B/Yamagata was the most frequently detected subtype during the 2014 season in the Philippines and A(H1N1) was the most frequently detected during the 2015/2016 season in the European countries.

**Table 3:** Circulation of influenza strains by region and season and attack rates

Subtype/ lineage	Confirmed influenza episodes, n (%)						
	South Africa 2014	Philippines 2014	Philippines 2015	European countries ^a^ 2014/2015	European countries ^a^ 2015/2016	Total	
	*N* = 17	*N* = 93	*N* = 45	N = 37	*N* = 71	*N* = 263	Attack rate ^b^, % (95% CI)
A(H1N1)	0 (0.0)	0 (0.0)	2 (4.4)	2 (5.4)	45 (63.4)	49 (18.6)	2.2 (1.6; 2.8)
A(H3N2)	16 (94.1)	23 (24.7)	43 (95.6)	24 (64.9)	1 (1.4)	107^c^ (40.7)	4.6 (3.7; 5.5)
B/Yamagata	1 (5.9)	53 (57.0)	0 (0.0)	8 (21.6)	0 (0.0)	62 (23.6)	2.8 (2.1; 3.5)
B/Victoria	0 (0.0)	0 (0.0)	0 (0.0)	0 (0.0)	21 (26.9)	21 (8.0)	1.0 (0.5; 1.4)
Not subtyped	0 (0.0)	17 (18.3)	0 (0.0)	3 (8.1)	4 (5.6)	24 (9.1)	1.1 (0.7; 1.5)

^a^France, Greece, Italy, Spain, and Romania

^b^Calculated as the number of participants with at least one episode of confirmed influenza divided by the total number of participants (*N* = 2210)

^c^107 episodes in 102 participants

### Burden of influenza

Grade 3 fever was reported for about one-quarter (24.3%) of the 263 confirmed influenza episodes (Table 4). Less than 10% of the confirmed influenza episodes were associated with acute lower respiratory infection (8.7%) or AOM (6.1%). Influenza was associated with pneumonia in 1.9% of the confirmed episodes.

**Table 4:** Occurrence of grade 3 symptoms, complications, and healthcare use associated with confirmed influenza episodes

	Confirmed influenza episodes	Proportion of episodes
Laboratory-confirmed influenza associated with:	*N* = 263	(%)
Grade 3 symptoms		
Grade 3 fever ^a^	63	24.3
≥ 1 grade 3 influenza-like illness symptom ^b^	8	3.0
Complications		
Acute otitis media	16	6.1
Acute lower respiratory infection	23	8.7
Pneumonia	5	1.9
Medication		
Any	257	97.7
Antibiotics	109	41.4
Antipyretic or analgesic medication ^c^	245	93.2
Antitussives	8	3.0
Bronchodilators	36	13.7
Expectorants	10	3.8
Mucolytic agents	47	17.9
Healthcare use		
Outpatient visit	150	57.0
Inpatient hospitalisation	3	1.1
Intensive care unit admission	0	0.0

^a^> 39.5 °C for subjects aged < 24 months and ≥ 39.0 °C for subjects aged ≥24 months

^b^Cough, nasal congestion, rhinorrhoea, pharyngitis, otitis, vomiting, or diarrhoea preventing daily activities

^c^Includes non-steroidal anti-inflammatory drugs

Participants received medication during nearly all confirmed influenza episodes (97.7%). For 93.2% of confirmed influenza episodes, antipyretic or analgesic medication was taken. For 41.4% of the confirmed influenza episodes, antibiotics were prescribed and taken. Antipyretic or analgesic medication, antitussives, bronchodilators, expectorants, and mucolytic agents were each prescribed in fewer than 20% of the confirmed influenza episodes. Medication use by region is described in Additional file 2: Table S1.

More than half of the confirmed influenza episodes (57.0%) resulted in one or more outpatient visits. Few episodes resulted in overnight hospitalisation (*n* = 3; 1.1%). No children with confirmed influenza were admitted to an intensive care unit.

## Discussion

Although the WHO recommends including young children as a target group for influenza vaccination, many countries do not include them in their recommendations [5], probably because severe influenza has not been considered a problem for children without underlying high-risk conditions [6]. In this study we showed that 11.5% of 2210 healthy unvaccinated children 6 to 35 months of age contracted influenza during the influenza season. In many of these children, infection with influenza virus resulted in high fever, complications, medical visits, and use of antibiotics.

We found that more than one-third of influenza episodes were associated with grade 3 fever. AOM, also common in cases of influenza [9], was reported for 6.1% of influenza episodes. This is lower than that found in another study of children aged < 3 years (39.7%) [10] but close to rates reported in studies of children aged < 2 years (6.4%) and 2 to 4 years (4.9%) [11]. Acute lower respiratory infections were documented for 8.7% of influenza episodes, which is lower than the combined rate of 13% for children < 5 years of age reported in a meta-analysis by Nair et al. [12]. Finally, influenza was associated with pneumonia in just under 2% of episodes.

The rates of medication use, outpatient visits, and hospitalisations in the study generally agreed with a systematic review by Antonova et al. [3] who reported that 76 to 99% of children aged < 18 years with laboratory-confirmed influenza received antipyretic or other medications for symptomatic relief and that 0.3 to 20% were hospitalised. Importantly, antibiotics were prescribed for more than 40% of the confirmed influenza episodes. A few of these antibiotic prescriptions could have been for influenza-associated AOM or acute lower respiratory infection, which were observed in 15% of influenza cases. We did not find other reports describing antibiotic use in young children with ILI, although the systematic review by Antonova et al. reported that influenza is associated with antibiotic prescriptions in 7 to 55% of cases in European children aged < 18 years [3]. A retrospective analysis of the US Impact National Benchmark Database from 2005 to 2009 found that antibiotics were prescribed in about 22% of all patients with influenza and were used inappropriately in 79% of the cases because of an absence of secondary infection or comorbidity [13]. Thus, unnecessary antibiotic use in influenza appears to be a continuing problem and may be contributing to the spread of antibiotic-resistant bacteria [13, 14].

Strain circulation differed between consecutive seasons in the same region. For example, in the included European countries, A(H3N2) dominated during the 2014/2015 influenza season and A(H1N1) dominated during the 2015/2016 season. In the Philippines, B/Yamagata dominated during the 2014 season, whereas A(H3N2) dominated during the 2015 season. In both seasons in the European countries, the B lineage recommended by the WHO for the trivalent vaccine [15, 16] did not match with the dominant circulating B lineage. Although the study included relatively few confirmed influenza episodes, strain circulation in this study population generally agreed with the WHO seasonal reports [15–18].

A unique characteristic of our analysis is that the data resulted from active surveillance during a randomised, placebo-controlled vaccine efficacy trial. This favours systematic and complete data collection, and therefore would have reduced the chance of missing ILI episodes. Although active surveillance could have increased the rate of ILI compared to passive surveillance, the overall influenza attack rate (11.5%) was consistent with the rate described in a 2014 meta-analysis (15.2% [95% confidence interval, 11.4 to 18.9]) for unvaccinated children aged < 18 years [19]. However, this overall attack rate is substantially lower than that described by the WHO (20 to 30% for children) [2]. Similarly, the higher attack rate in the European countries (20.5%) compared to the other countries (6.7%–9.3%) cannot be explained by the surveillance procedures used to identify ILI cases, since these were identical in all participating countries. The burden of influenza between countries can vary widely and is determined by a number of factors including the characteristics of circulating viruses, and the timing and severity of the season [4]. Our study included countries located in different regions with different latitudes, climates, and influenza seasonality that may partly explain the differences between the influenza attack rates observed. For example, in Europe, the 2014–2015 season was particularly severe [20], which could partly explain the higher attack rate reported compared to other regions.

An important limitation of this analysis is that the population was selected for the phase III clinical trial and may not have been representative of the wider population in the included regions. Specifically, the trial included children without comorbidities who were presumed to be immunologically naïve to influenza. The inclusion of only children without comorbidities may explain why the attack rate was lower in this analysis than reported by the WHO [2] and in the 2014 meta-analysis [19].

A final limitation is that Honduras and the Dominican Republic were not included in this analysis because participants in this region had been recruited outside the influenza season – which is often difficult to define in the tropics and subtropics [21] – resulting in no confirmed influenza cases.

## Conclusions

With data available from the placebo arm of a large clinical trial [7], we confirmed that healthy young children are susceptible to influenza and that, in many cases, influenza infection in young children is associated with severe symptoms, frequent healthcare use, and inappropriate antibiotic use. These findings should support efforts to enact recommendations by the WHO to include young children in national influenza vaccination policies.

## Additional file

Additional file 1: **Figure S1.** Participant flow diagram for the original study (PDF 12 kb)

Additional file 2: **Table S1.** Medication use associated with confirmed influenza episodes by region (PDF 8 kb)

Additional file 3 Ethics Committees that approved the original study (PDF 139 kb)

## Abbreviations

AOM: Acute otitis media
ILI: Influenza-like illness
WHO: World Health Organization
