# Supplementary figures and images for: Epidemiology and burden of influenza in healthy children aged 6 to 35 months: analysis of data from the placebo arm of a phase III efficacy trial
Source: BMC Infect Dis. 2019 Apr 4;19:308. doi: 10.1186/s12879-019-3920-8 (PMC6449994; doi:10.1186/s12879-019-3920-8)

**Supplementary Figure 1: Participant flow diagram for the original study**

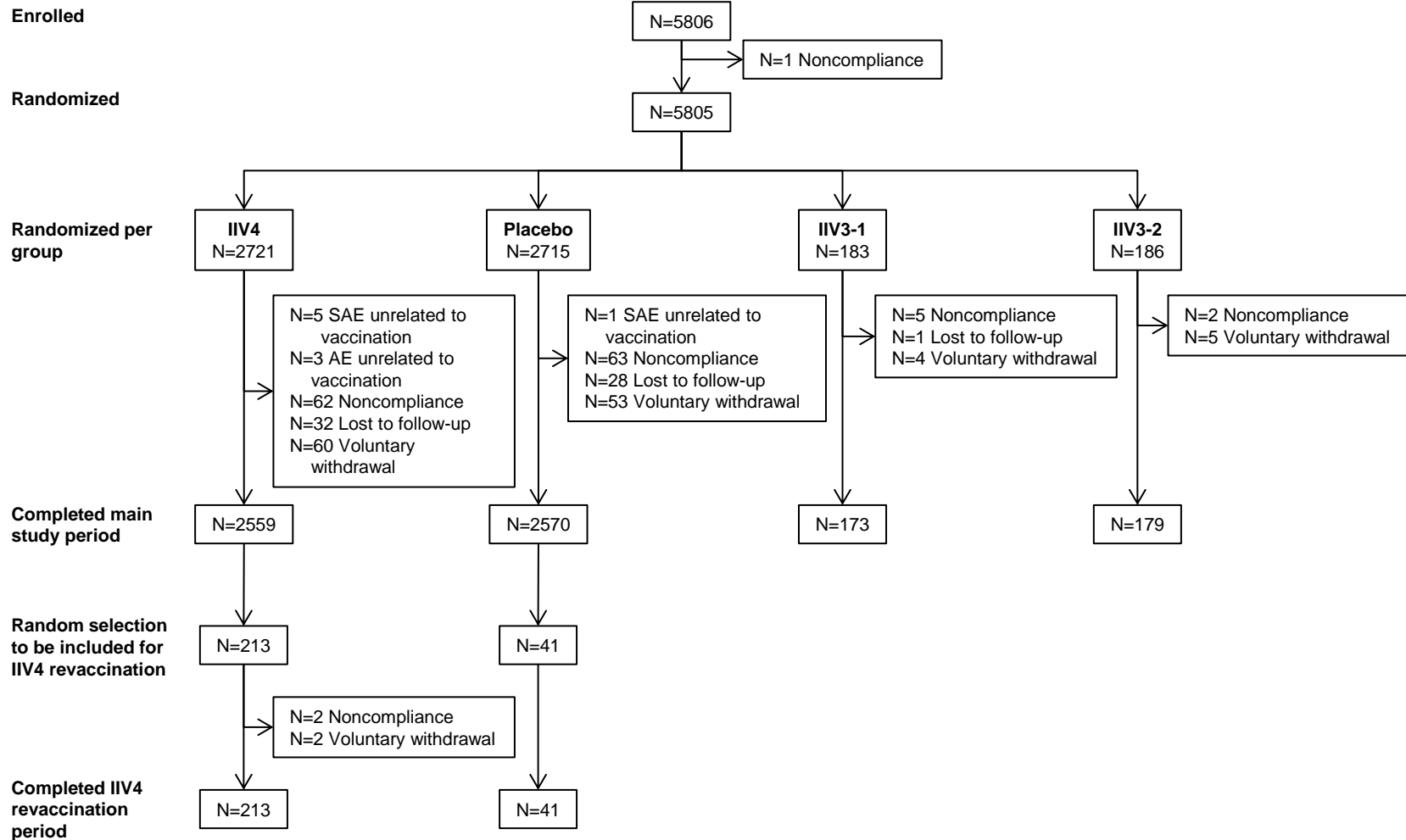

Supplement: Supplementary file 1 — Figure S1. Participant flow diagram for the original study (PDF 12 kb) [file 12879_2019_3920_MOESM1_ESM.pdf]
