# Supplementary material for: Epidemiology and burden of influenza in healthy children aged 6 to 35 months: analysis of data from the placebo arm of a phase III efficacy trial
Source: BMC Infect Dis. 2019 Apr 4;19:308. doi: 10.1186/s12879-019-3920-8 (PMC6449994; doi:10.1186/s12879-019-3920-8)
Supplement: Supplementary file 2 — Table S1. Medication use associated with confirmed influenza episodes by region (PDF 8 kb) [file 12879_2019_3920_MOESM2_ESM.pdf]

Supplementary Table 1. Medication use associated with confirmed influenza episodes by region

Laboratory-confirmed influenza associated with:	South Africa N=17	Philippines N=138	European countries [a] N=108
Any medication	16 (94.1)	137 (99.3)	104 (96.3)
Antibiotics	4 (23.5)	63 (45.7)	42 (38.9)
Pain medication [b]	16 (94.1)	130 (94.2)	99 (91.7)
Antitussives	2 (11.8)	1 (0.72)	5 (4.6)
Bronchodilators	1 (5.9)	24 (17.4)	11 (10.2)
Expectorants	0 (0.0)	6 (4.3)	4 (3.7)
Mucolytic agents	5 (29.4)	36 (26.1)	6 (5.6)

[a] France, Greece, Italy, Spain, and Romania

[b] Antipyretics, analgesics, non-steroidal anti-inflammatory drugs
