# Supplementary material for: Epidemiology and burden of influenza in healthy children aged 6 to 35 months: analysis of data from the placebo arm of a phase III efficacy trial
Source: BMC Infect Dis. 2019 Apr 4;19:308. doi: 10.1186/s12879-019-3920-8 (PMC6449994; doi:10.1186/s12879-019-3920-8)
Supplement: Supplementary file 3 — Ethics Committees that approved the original study (PDF 139 kb) [file 12879_2019_3920_MOESM3_ESM.pdf]

Ethics Committees that approved the original study

The original phase III randomised controlled clinical trial (EudraCT no. 2013-001231-51) was approved by the following independent ethics committees and institutional review boards:

- Research Institute for Health Sciences, University of the East Ramon Magsaysay Memorial Medical Center, 64 Aurora Boulevard, Barangay Doña Imelda, Quezon City, Philippines 1113.
- RITM Institutional Review Board, Research Institute for Tropical Medicine Filinvest Corporate City, 1781 Alabang, Muntinlupa, Philippines
- PCMC Institutional Review Board-Ethics Committee, PCMC Quezon Avenue corner, Agham Road, Quezon City, Metro Manila 1101, Philippines
- Mary Chiles General Hospital Ethics Review Board, Mary Chiles General Hospital, 667 Dalupan Sr (Formerly Gastambide) St., Sampaloc City of Manila, Metro Manila, Philippines
- De La Salle-Health Sciences Institute Independent Ethics Committee, Angelo King Medical Research Center, Pasong Lawin, Dasmariñas, Cavite 4114, Philippines
- University of the Philippines Manila Research Ethics Board, 2F Paz Mendoza Building, College of Medicine, UP Manila, 547 Pedro Gil Street, Ermita, 1000 Manila, Philippines
- Human Research Ethics Committee, Medical Wits Ethics Secretariat Office, Suite 189, Private Bag x2600, Houghton 2041, South Africa
- Comité de Ética en Investigación Biomédica. UIC. Facultad de Ciencias Médicas. UNAH, Blvd. Suyapa Facultad de Ciencias Médicas Universitaria, Tegucigalpa, Honduras
- Comité de Ética Independiente de la Fundación Dominicana de Infectología, INC. Abraham Lincoln #2 Esq. Ave. Independencia. Centro de los Héroes. Santo Domingo, Dominican Republic
- CPP Sud-Ouest et Outre Mer III, Service de Pharmacologie Clinique, Bat. 1A, Hôpital PELLEGRIN, Place Amélie Raba Léon, 33076 Bordeaux Cedex, France
- National Ethics Committee (NEC), 284 Mesogeion Avenue, 155 62 Cholargos, Athens, Greece
- Hospital Scientific Committee, University General Hospital “Attikon, 1, Rimini Street, P.C. 124 62, Chaidari, Athens, Greece
- Hospital Scientific Committee, “P. and A. Kyriakou” University Children Hospital, 2nd Department of Pediatrics, 11527Goudi, Athens, Greece
- Hospital Scientific Committee, University Hospital of Thessaloniki "Papageorgiou", Thessaloniki Ring Road, 56403 Nea Efkarpia, Thessaloniki, Greece
- Hospital Scientific Committee, University Hospital of Thessaloniki, “Hippokration”, Konstantinoupoleos 49, Thessaloniki 54642, Greece
- Hospital Scientific Committee, "Agia Sofia" University Children Hospital ", Infectiuos Diseases Dpt, Thivon & M. Asias, 11527 Goudi, Athens, Greece

- Comitato Etico Regione Liguria-SEZ.3 c/o IRCCS- A.O.U. San Martino-IST Istituto Nazionale Per La Ricerca Sul Cancro, Largo Rosanna Benzi 10, 16132 Genova, Italy
- Comitato di Etica della Fondazione IRCCS Ca' Granda - Ospedale Maggiore Policlinico, Via Francesco Sforza n. 28, 20122 Milano, Italy
- Comitato Etico Interaziendale dell’Azienda Ospedaliero Universitaria Maggiore della Carità di Novara, Corso Mazzini, 18, 28100 Novara, Italy
- Comitato Etico Per La Sperimentazione Clinica Della Provincia Di Padova, Via Giustiniani, 2, 35128 Padova, Italy
- Spettabile, Comitato Etico, Catania , Via Santa Sofia 78, 95123 Catania, Italy
- REFERENCE EC, CEIC Área 5 - Hospital Universitario La Paz, Paseo de la Castellana, 261, 28046 Madrid, Spain
- CEIC Fundacio Investigacio en Atencio Primaria Jordi Gol i Gurina - IDIAP Jordi Gol, Gran Via de les Corts Catalanes, 587 àtic, 08007 Barcelona, Spain
- CEIC Área 10 - Hospital Universitario de Getafe, Ctra. De Toledo, km. 12,500, 28905 Getafe, Spain
- CEIC Malaga Nordeste, Pabellon A. Hospital General, 7 planta, 29010 Malaga, Spain
- CEIC Hospital Universitario De Elda, Ctra.Elda-Sax, Ptda La Torreta, 03600 Elda, Spain
- CEIC SERGAS, Comité Ético de Investigación Clínica de Galicia, Subdirección Xeral de Farmacia e Produtos Sanitarios. Conselleria de Sanidade, C/ San Lázaro, s/n, 15703 Santiago de Compostela, Spain
- CEIC Hospital Universitari de Tarragona Joan XXIII, Doctor Mallafré Guasch, 4, 43007 Tarragona, Spain
- CEIC Dirección General de Salud Pública y Centro Superior de Investigación en Salud Pública, Avda. Cataluña, 21, 46020 Valencia, Spain
- CEIC Hospital Universitario Virgen del Rocíom, Avda. Manuel Siurot, s/n. Edificio de Laboratorios - Planta 6ª , 41013 Sevilla, Spain
- CEIC Área 7 - Hospital Clínico San Carlos de Madrid, s/n - Ciudad Universitaria, 28040 Madrid, Spain
- National Bioethics Commission For Medicines and Medical Devices, 19-21 Stefan cel Mare Blvd, Bucharest 020125, Romania
